# New trends and hotspots in Sepsis-associated encephalopathy research: a bibliometric and visualization analysis

**DOI:** 10.3389/fnagi.2026.1780681

**Published:** 2026-04-01

**Authors:** Younian Wang, Lin Zheng, Fan Ye

**Affiliations:** 1 Hubei University of Arts and Science, Xiangyang, China; 2 Department of Anesthesiology, Xiangyang Central Hospital, Affiliated Hospital of Hubei University of Arts and Science, Xiangyang, China; 3 Institute of Neuroscience and Brain Science, Xiangyang Central Hospital, Affiliated Hospital of Hubei University of Arts and Science, Xiangyang, China

**Keywords:** bibliometrics, CiteSpace, clinical translation, sepsis-associated encephalopathy, visualization analysis

## Abstract

**Objective:**

To systematically map the global and domestic research landscape of sepsis-associated encephalopathy (SAE) and to identify current hotspots and future trends.

**Methods:**

Relevant articles published from 1 January 2000, to 1 August 2025 were retrieved from the Web of Science (WoS) Core Collection and the China National Knowledge Infrastructure (CNKI). Bibliometric software including CiteSpace V6.3. R1, VOSviewer V1.6.20 and Bibliometrix R V4.1.3 were employed to extract and visualize annual publications, distribution of research fields, countries/regions, authors, institutions, average citations per publication and keywords.

**Results:**

A total of 1,097 articles were included in this study. Over the past 25 years, the annual articles and annual citations in the field of SAE have shown an overall upward trend. Among various sepsis-related organ complications, SAE has undergone rapid development and gained an increasingly prominent research status. Studies in this field are characterized by multidisciplinary integration, involving neuroscience, immunology, pharmacology and pharmacy, emergency medicine, neurology, and other disciplines. China ranks first in publication volume, followed by the United States and Brazil. Tianjin Medical University is the most productive institution, while Nanjing University holds the highest average citations per paper. Core research groups led by “Xie Keliang” and “Yang Jianjun” have been formed at home and abroad. Burst keyword analysis shows a shift from early mechanistic terms such as “nitric oxide synthase”, “cerebral blood flow” and “tumor necrosis factor” to recent intervention-oriented terms including “cells”, “inhibition”, “treatment” and “electroacupuncture”, indicating a transition from basic mechanism exploration to clinical application and targeted intervention.

**Conclusion:**

SAE research continues to gain momentum. Future work should focus on the neuroinflammation-blood–brain barrier axis. Researchers may explore interactions among microglial activation, oxidative stress, and barrier damage, as well as the role of the vagal-immune metabolic pathway. Within a consensus-based framework, efforts can advance new prevention and treatment strategies. These strategies may combine multi-organelle targeted inhibition with multi-component interventions from traditional Chinese medicine (TCM).

## 1 Introduction

Sepsis is a life-threatening organ dysfunction syndrome caused by a dysregulated host response to infection and remains a leading cause of morbidity and mortality in intensive care units worldwide, with core pathological features involving the cascading activation of molecular mechanisms including endothelial dysfunction, excessive formation of neutrophil extracellular traps (NETs), imbalanced macrophage polarization, and disturbances in the coagulation-fibrinolysis system, all of which collectively drive disease progression and organ injury (Barkat et al., 2025; Catarina et al., 2021; Liu et al., 2022). Sepsis-associated encephalopathy (SAE), a diffuse cerebral dysfunction triggered by sepsis, stands as one of the most common neurological complications in intensive care settings, with incidence rates ranging from 50 to 70% (Li et al., 2026; Mazeraud et al., 2020). Its clinical manifestations vary from mild consciousness disturbances to severe coma, encompassing a spectrum of neuropsychiatric symptoms that significantly elevate patients’ mortality risk and potential for long-term cognitive impairment (Sonneville et al., 2023). Studies have reported that the mortality rate in patients with severe SAE may approach 50–70% (Li et al., 2026). Recent clinical investigations have further demonstrated that sepsis patients with altered mental status have a mortality rate of 49%, which is markedly higher than the 26% in those without neurological involvement (Liu et al., 2025). Such variations between studies may be attributed to differences in diagnostic criteria, study populations, and geographic regions. Although SAE is recognized as a crucial prognostic factor in sepsis mortality (Li et al., 2020; Mazeraud et al., 2020), conventional perspectives have regarded it as merely a central nervous system manifestation of systemic inflammation. However, in-depth investigations reveal that SAE involves more complex pathological processes, including excessive neuroinflammation, mitochondrial dysfunction, blood–brain barrier disruption, and immunosuppression (Hong et al., 2023; Ren et al., 2020). These pathological processes interact with one another to form a vicious cycle that eventually leads to neuronal damage and cognitive impairment. Notably, while these complex pathological changes increase the difficulty of treating SAE, they also provide clear targets for precision therapy. Elucidating the core pathological mechanisms provides critical scientific support for developing targeted therapeutic strategies and designing multi-target combination therapies.

In recent years, remarkable progress has been made in SAE research, forming a multidimensional research framework encompassing pathological mechanisms, biomarkers, clinical diagnosis and treatment, and prognostic evaluation. However, systematic, multi-database integrated analyses of the overall research status, developmental trends, and emerging hotspots in this field remain relatively scarce. Although Zhang et al. (2022) conducted a bibliometric analysis of publication trends, international collaborations, thematic evolution, and other fundamental landscape characteristics of SAE research from 2001 to 2021 using the Web of Science (WoS) database, existing bibliometric studies are still largely confined to single English-language databases. These studies typically adopt narrow and outdated search timeframes, which fail to fully capture both long-term trajectories and the latest research trends. Furthermore, there is a lack of systematic integration of the China National Knowledge Infrastructure (CNKI) database and comparative analyses between domestic and international research. This deficiency impedes a comprehensive understanding of the global research landscape, collaborative networks, and developmental disparities in the field of SAE. Meanwhile, Qi et al. (2025) restricted their analysis to the single domain of biomarkers, thus failing to encompass the full research landscape of SAE.

To address these gaps, we conducted a bibliometric analysis of SAE research spanning multiple dimensions, time periods, and languages by integrating WoS and CNKI. WoS captures global trends as the leading international database, while CNKI reflects domestic research characteristics. This dual-database approach allows for a balanced comparison of international and domestic scholarship. We examined publication trends, evolving hotspots, and emerging frontiers; compared research discrepancies across regions; and mapped the field’s knowledge trajectory to inform future studies.

## 2 Materials and methods

### 2.1 Data collection

This study employed bibliometric methods to systematically outline the research framework, trends and hotspots of SAE, providing a reference for subsequent investigations.

The WoS Core Collection was used as the international data source. Topic (TS) searches were performed with a comprehensive set of English terms for SAE. The search string was: TS = (“Sepsis-Associated Encephalopathy” OR “Sepsis Associated Encephalopathy” OR “Septic Associated Encephalopathy” OR “Septic-Associated Encephalopathy” OR “Endotoxin-related encephalopathy” OR “LPS-induced encephalopathy” OR “CLP-induced encephalopathy”). Only English articles and reviews published from January 1, 2000, to August 1, 2025 were included. Duplicates, retracted papers and irrelevant records were removed via the WoS built-in deduplication tool and manual verification, which yielded 720 valid records. The CNKI was used as the supplementary Chinese database. TS searches were performed from January 1, 2000, to August 1, 2025 using English translations of standardized Chinese SAE terms. The search string was: TS = (“Sepsis-Associated Encephalopathy” OR “Sepsis Encephalopathy” OR “Septic Encephalopathy” OR “Septicemia-Associated Encephalopathy” OR “septicemia Encephalopathy” OR “Septicemic Encephalopathy”). A total of 643 publications were initially retrieved. After excluding 236 dissertations, 17 conference papers and 13 non-academic documents, this step retained 377 eligible Chinese publications.

**Figure 1:**
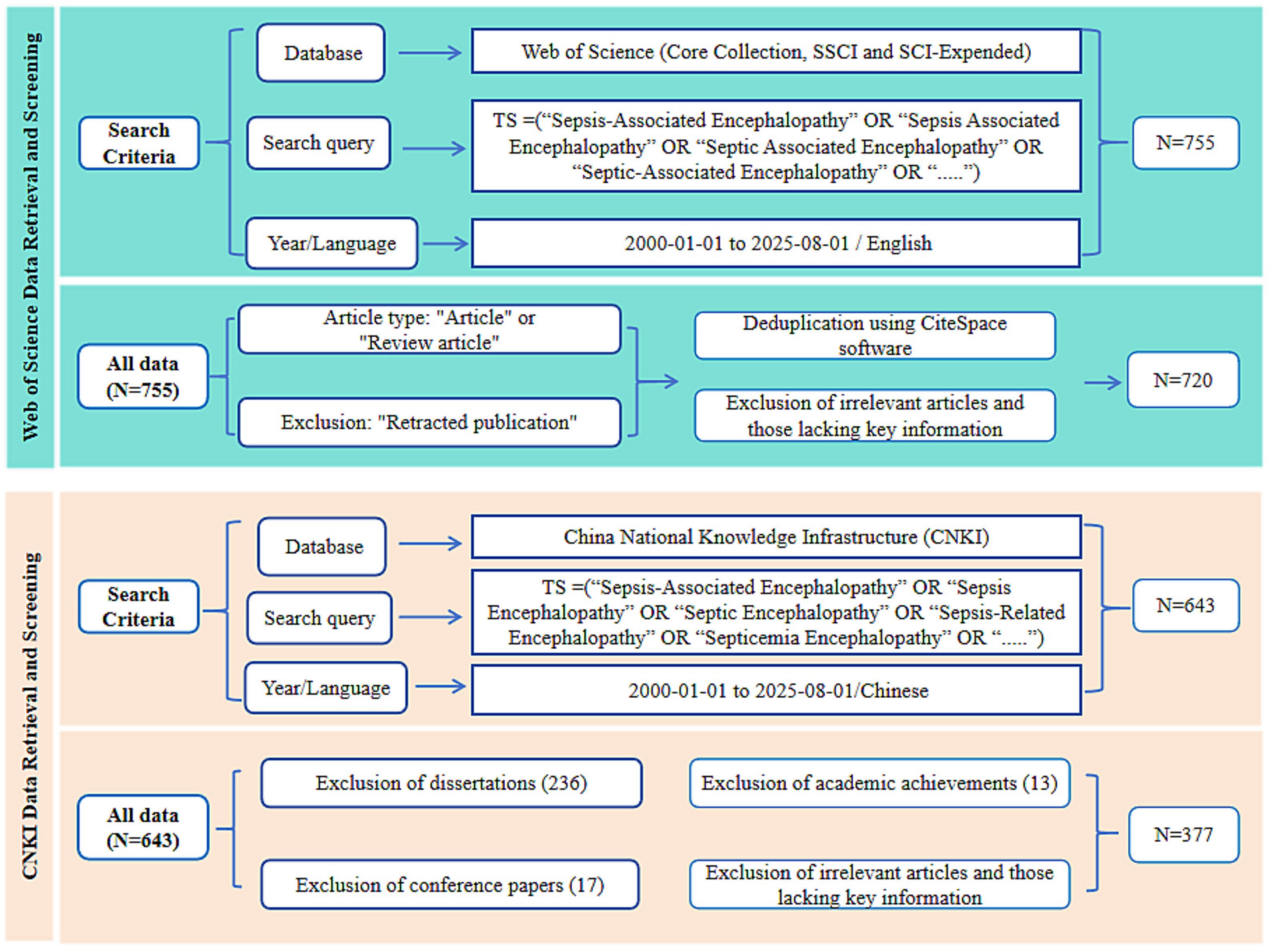
Flowchart of literature search and screening for SAE research.

To help international readers follow our approach, we translated all Chinese search terms into standard English equivalents. These include the official name for SAE, its common abbreviations, and other variants widely used in the literature. Multiple synonyms were used to capture as many relevant studies as possible and reduce retrieval bias. We then integrated and screened studies from both English and Chinese sources. Figure 1 shows the full selection process and data sources, which together ensure the reliability of our dataset.

### 2.2 Data analysis

In this study, data analysis followed a four-stage bibliometric workflow: data cleaning, network construction, evolution tracking, and visualization. First, full-text records from the WoS Core Collection and CNKI were batch-imported into CiteSpace 6.3. R1. Data cleaning encompassed deduplication, synonym merging, and institutional standardization to generate a refined dataset. Synonym merging was conducted strictly in accordance with the Medical Subject Headings (MeSH), the English-Chinese Medical Vocabulary (4th Edition), and authoritative reviews in the SAE field. For example, synonymous expressions such as “Sepsis-Associated Encephalopathy,” its abbreviation SAE, and “Septic Encephalopathy” were merged. Institutional standardization adhered to the principles of “unified name, standardized abbreviation, and clear affiliation,” referring to official institutional names and national naming standards. For instance, “Jilin University First Hospital” and “The First Hospital of Jilin University” were unified as “The First Hospital of Jilin University,” and “Harvard Med Sch” was standardized to “Harvard Medical School.” Additionally, to address author homonymy encountered during data processing, we distinguished authors by integrating information on their affiliated institutions, research areas, and co-authorship networks, thereby avoiding misattribution and statistical bias.

Subsequently, we constructed a country-keyword bipartite co-occurrence network in VOSviewer 1.6.20. Node size scaled with either publication volume (country nodes) or occurrence frequency (keyword nodes); larger nodes thus denoted greater research output or higher term prevalence, respectively. Inter-node edges encoded collaboration intensity, with thickness proportional to cooperation frequency.

To advance the analysis, CiteSpace was deployed across 2000–2025 with a 1-year time slice. Keyword clusters were extracted using the Log-Likelihood Ratio (LLR) algorithm, and a Timezone View was mapped to demonstrate the evolutionary path of the research domain (Liu et al., 2024). Burst detection was simultaneously run to flag keywords with citation surges, thereby tracing the temporal shifts of emerging research frontiers.

Spatial patterns of international collaboration were visualized via the countryCollaborationMap function in Bibliometrix R 4.1.3, which generated a cartogram weighted by normalized collaboration frequencies. Finally, we conducted descriptive statistical analyses on annual publication volumes, country distributions, author productivity and institutional contributions. All statistical procedures were implemented in WPS Office.

## 3 Results

### 3.1 Fundamental quantitative data

A total of 1,398 publications on SAE were retrieved in this study, including 755 from the WoS Core Collection and 643 from the CNKI database. After screening according to strict inclusion and exclusion criteria (Figure 1), 1,097 articles were finally included. Of these, 720 were from the WoS Core Collection (607 original articles and 113 reviews), and 377 were from CNKI (316 original articles and 61 reviews). The included studies involved 4,968 authors and yielded 3,360 keywords.

### 3.2 Quantitative analysis of publications

From 2000 to 2024, the annual number of publications on SAE rose steadily. Marked disparities in volume and growth patterns were evident for original and review articles. Original articles dominated the publications (71.47% in CNKI and 83.66% in the WoS Core Collection). Review articles, by contrast, increased after 2011, accelerating markedly thereafter. This trajectory traces SAE’s rise from a marginal clinical issue to a core research direction in critical care medicine.

According to publication trends, this period can be divided into three stages with distinct research drivers and characteristics. Initial stage (2000–2010): Research during this stage was driven by the urgent clinical demand for identifying septic encephalopathy. Previously, sepsis-associated cognitive dysfunction was often attributed to metabolic disturbances or drug side effects, with no established independent diagnostic criteria (Gofton and Young, 2012). The 2001 International Sepsis Definitions Conference included altered consciousness as a key feature in sepsis diagnosis, spurring early exploratory studies (Levy et al., 2003). During this period, the WoS Core Collection indexed 25 SAE publications (21 original articles, 4 reviews), while CNKI indexed 16 (11 original articles, 5 reviews). Original articles dominated both databases, with reviews remaining scarce. This pattern indicates the field was still in an exploratory phase, with review articles lagging far behind empirical research. Preliminary development stage (2011–2017): In contrast to descriptive studies in the initial stage, research in this stage shifted toward mechanistic exploration and biomarker validation. The 2016 Sepsis-3 international consensus formally integrated acute brain dysfunction into the evaluation of organ failure through the SOFA score, substantially stimulating SAE-related research (Singer et al., 2016; Vincent et al., 1996). Concurrently, the Surviving Sepsis Campaign guidelines emphasized the monitoring and assessment of delirium and central nervous system function (Rhodes et al., 2017). During this stage, the WoS Core Collection indexed 93 SAE publications (78 original articles and 15 review articles), and CNKI indexed 87 (64 original articles and 23 review articles). The volume of Chinese and English publications gradually converged. Original articles remained the dominant research output, while the number of review articles increased, reflecting growing emphasis on systematic summaries of research advances in the field. Rapid development stage (2018–2024): This stage displayed two prominent features: an exponential increase in publication output and a shift in research paradigm. The WoS Core Collection indexed a total of 494 publications (413 original articles and 81 review articles), with an annual average of 70.57 publications and a peak in 2024. Correspondingly, CNKI indexed 251 publications (178 original articles and 73 review articles), with an annual average of 35.86 publications and a peak in 2022. The discrepancy in peak years (2024 vs. 2022) between the two databases reflects differences in publication cycles and indexing delays; divergent peer-review schedules between domestic and international journals resulted in distinct publication peaks for COVID-19-related studies. More significantly, research focus changed from “phenomenon description” to “precision intervention”: international research has been dominated by multi-omics integration and the development of machine learning-based prognostic models, whereas domestic studies have focused on clinical pathway optimization. The marked increase in review articles reflects a growing demand for systematic summaries of research progress and guidance for future directions in the field. During the COVID-19 pandemic, acute brain dysfunction and long-term cognitive sequelae (Long COVID) in critically ill patients attracted widespread attention. Their underlying mechanisms overlapped strongly with those of SAE, further fueling research interest in this field (Ellul et al., 2020; Taquet et al., 2021). The surge in international publications after 2021 was closely associated with neurological complications of Long COVID, whereas the 2020–2021 peak in domestic publications mainly reflected summaries of early clinical experience.

**Figure 2:**
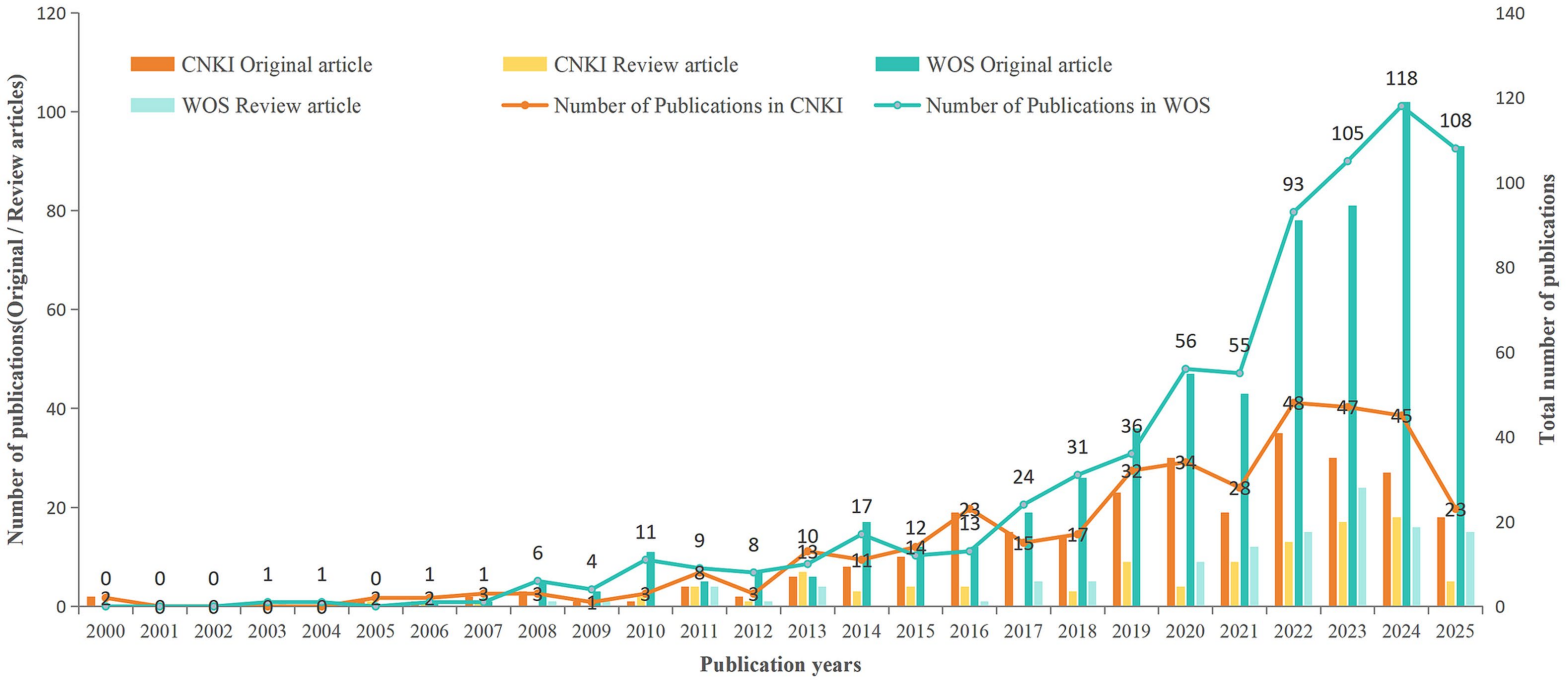
Annual publications of SAE research in the CNKI and WoS databases.

Overall, publication outputs from both databases exhibited continuous growth, but their driving forces and academic ecosystems differed: the WoS Core Collection demonstrated the consistent leadership and cutting-edge orientation of international research, while CNKI reflected the rapid responsiveness and clinical applicability of domestic studies (Figure 2). As the primary carrier of original research, original articles represented the main source of knowledge expansion in the field. Review articles served to integrate research progress and distill academic consensus. The two types complemented each other to form a complete academic framework for SAE research, and their distinct citation patterns further attested to their unique academic contributions.

### 3.3 Distribution of research fields

Table 1 reveals that SAE research spans multiple disciplines, with marked distributional disparities and distinct disciplinary profiles between the two databases.

**Table 1:** Top 10 major research fields of SAE research.

Rank	WoS major research fields	Percentage (%)	CNKI major research fields	Percentage (%)
1	Neuroscience	34.44	Emergency Medicine	35.28
2	Immunology	16.53	Clinical Medicine	31.13
3	Pharmacology Pharmacy	15.14	Neurology	16.68
4	Biochemistry Molecular Biology	10.14	Pediatrics	5.15
5	Experimental Medicine	10.14	Traditional Chinese Medicine	3.15
6	Critical Care Medicine	8.19	Chinese Materia Medica	2.38
7	Cell Biology	7.50	Surgery	2.00
8	Clinical Neurology	7.36	Gastroenterology	1.31
9	Medicine General Internal	3.89	Pharmaceutical Sciences	0.69
10	Multidisciplinary Science	3.89	Basic Medical Sciences	0.61

In the WoS Core Collection, SAE-related studies are oriented toward basic science. Neuroscience accounts for the largest share (34.44%), representing the core research domain, followed by Immunology (16.53%) and Pharmacology and Pharmacy (15.14%). Collectively, these three disciplines account for over 66% of publications, with additional contributions from Biochemistry Molecular Biology, Experimental Medicine, Critical Care Medicine, and related fields, reflecting an international emphasis on fundamental mechanisms and pharmacological interventions for SAE.

By contrast, studies indexed in CNKI are clinically oriented. Emergency Medicine constitutes the largest proportion (35.28%), followed by Clinical Medicine (31.13%); together these two disciplines comprise over 66% of the literature. Neurology contributes 16.68%, bringing the cumulative share of these three clinical disciplines to over 83%. Additional fields, including Traditional Chinese Medicine, Chinese Materia Medica, and Surgery, are also represented, underscoring the tight integration of domestic research with clinical practice.

This distributional pattern not only underscores the highly interdisciplinary nature of SAE research but also reveals divergent disciplinary orientations and research priorities between domestic and international SAE studies.

### 3.4 National and institutional analysis

**Figure 3:**
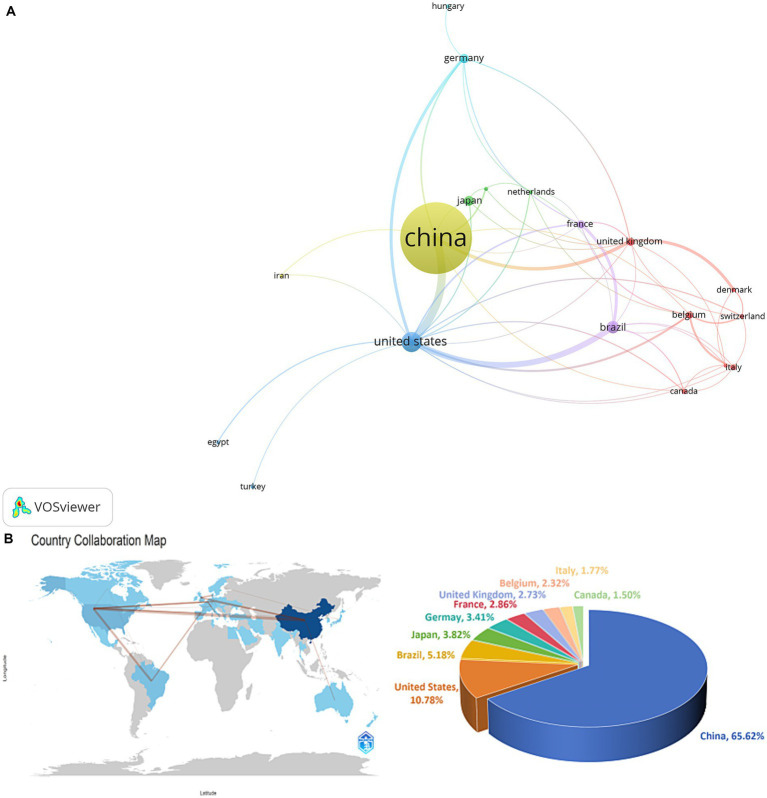
**(A)** National collaboration network of SAE research. **(B)** Global collaboration heatmap and publication distribution of SAE research.

Publications on SAE research were retrieved from 41 countries. As shown in Table 2 and Figure 3B, China contributed 481 articles, accounting for 65.62% of the total, followed by the United States (79, 10.78%) and Brazil (38, 5.18%). Citation performance differed substantially across countries. Canada ranked first with 102.64 citations per article, while France, Brazil and Germany exceeded 50. Although China led in publication output, its citation rate of 17.17 reflected a gap in research impact. This may reflect several factors: research on SAE in China started late; most studies focused on clinical observation and practical applications rather than high-citation topics such as mechanism exploration, multi-omics analysis, and prognostic model construction; and many domestic findings were published in Chinese-language journals, limiting international visibility and dissemination. These factors contributed to the lower citation performance of Chinese publications compared with developed countries in Europe and North America.

**Table 2:** Top 10 countries by SAE research publications.

Rank	Country	Number of publications	Total citations	Citations per publication	Percentage (%)	Total link strength
1	China	481	8,260	17.17	65.62	33
2	United States	79	3,247	41.10	10.78	51
3	Brazil	38	2013	52.97	5.18	24
4	Japan	28	376	13.43	3.82	4
5	Germany	25	1,253	50.12	3.41	13
6	France	21	1,354	64.48	2.86	16
7	United Kingdom	20	895	44.75	2.73	14
8	Belgium	17	555	32.65	2.32	12
9	Italy	13	348	26.77	1.77	7
10	Canada	11	1,129	102.64	1.50	6

We mapped national and international collaboration networks using VOSviewer and Bibliometrix R (Figure 3B). Network centrality reflected research output: China, the United States, Brazil and Japan displayed the largest nodes. China collaborated most intensively with the United States, the United Kingdom, Germany and France (link strength: 33), whereas the United States maintained broader ties (link strength: 51) spanning China, Brazil, Germany and France. Among the top 10 countries, only China and Brazil represented developing economies; the other eight were developed countries.

### 3.5 Author analysis

VOSviewer identified 4,968 authors involved in SAE research (Table 3). In the WoS Core Collection, 13 of the 15 most productive authors originated from China, evidence of China’s strong cluster advantage in this field. China’s Xie Keliang ranked first with 20 publications, 19.00 citations per publication, and a total link strength of 22. Sharshar Tarek from France and Dal-Pizzol Felipe from Brazil each published 10 publications and tied for tenth place; their average citations per publication stood at 94.50 and 82.70, evidence of outstanding academic influence. Ji Muhuo and Yang Jianjun from China shared fourth place with 40.69 citations per paper, a mark of improved domestic research quality.

**Table 3:** Top 15 authors by SAE research publications.

Rank	WoS database						CNKI database			
	Author	Country	Publications	Total Citations	Citations per publication	Total link strength	Author	Publications	Total link strength	Affiliation
1	Xie, Keliang	China	20	380	19.00	22	Yang, Jianjun	10	12	The First Affiliated Hospital of Zhengzhou University
2	Yu, Yonghao	China	17	359	21.12	11	Ji, Huomu	8	12	Zhongda Hospital Affiliated to Southeast University
3	Zhang, Lina	China	17	240	14.12	5	Fang, Zongping	8	4	Xijing Hospital of Air Force Medical University
4	Zhao, Lina	China	15	155	10.33	21	Li, Xiaoliang	5	8	The First People’s Hospital of Zhengzhou, Affiliated to Henan University
5	Li, Yi	China	14	221	15.79	17	Zhang, Xijing	5	4	The First Affiliated Hospital of Air Force Medical University
6	Ji, Muhuo	China	13	529	40.69	13	Duan, Meili	5	0	Beijing Friendship Hospital, Capital Medical University
7	Yang, Jianjun	China	13	529	40.69	13	Jia, Min	4	8	Nanjing General Hospital of Nanjing Military Region
8	Si, Yanna	China	11	237	21.55	10	Xia, Chengde	4	7	Zhengzhou First People’s Hospital
9	Bao, Hongguang	China	11	236	21.45	10	Xiao, Hongtao	4	7	Zhengzhou First People’s Hospital
10	Sharshar, Tarek	France	10	945	94.50	2	Lin, Xinfeng	4	4	The First Affiliated Hospital of Guangzhou University of Chinese Medicine
11	Dal-pizzol, Felipe	Brazil	10	827	82.70	2	Zheng, Shuming	4	4	The First Affiliated Hospital of Guangzhou University of Chinese Medicine
12	Zhang, xijing	China	10	146	14.60	14	Liu, Qingquan	4	0	Beijing Hospital of Traditional Chinese Medicine, Capital Medical University
13	Huang, Li	China	9	308	34.22	5	Zhang, Li	4	0	Shengli Oilfield Central Hospital
14	Fang, zongping	China	9	125	13.89	14	Chen, Jun	4	0	Qingyuan People’s Hospital of Guangdong Province
15	Li, Yun	China	9	113	12.56	15	Lu, Weihua	4	0	Yijishan Hospital, The First Affiliated Hospital of Wannan Medical College

Overall, Chinese scholars led globally in publication volume, but their average citation performance still lags behind top international teams. In the CNKI database, the top three authors ranked by productivity are all domestic clinical experts: Yang Jianjun from the First Affiliated Hospital of Zhengzhou University, Ji Muhuo from Zhongda Hospital Affiliated to Southeast University, and Fang Zongping from Xijing Hospital of the Air Force Medical University. The first two scholars also exhibited the highest total link strengths, a clear indication that top-level hospitals in China are core hubs of SAE research.

### 3.6 Institutional analysis

**Figure 4:**
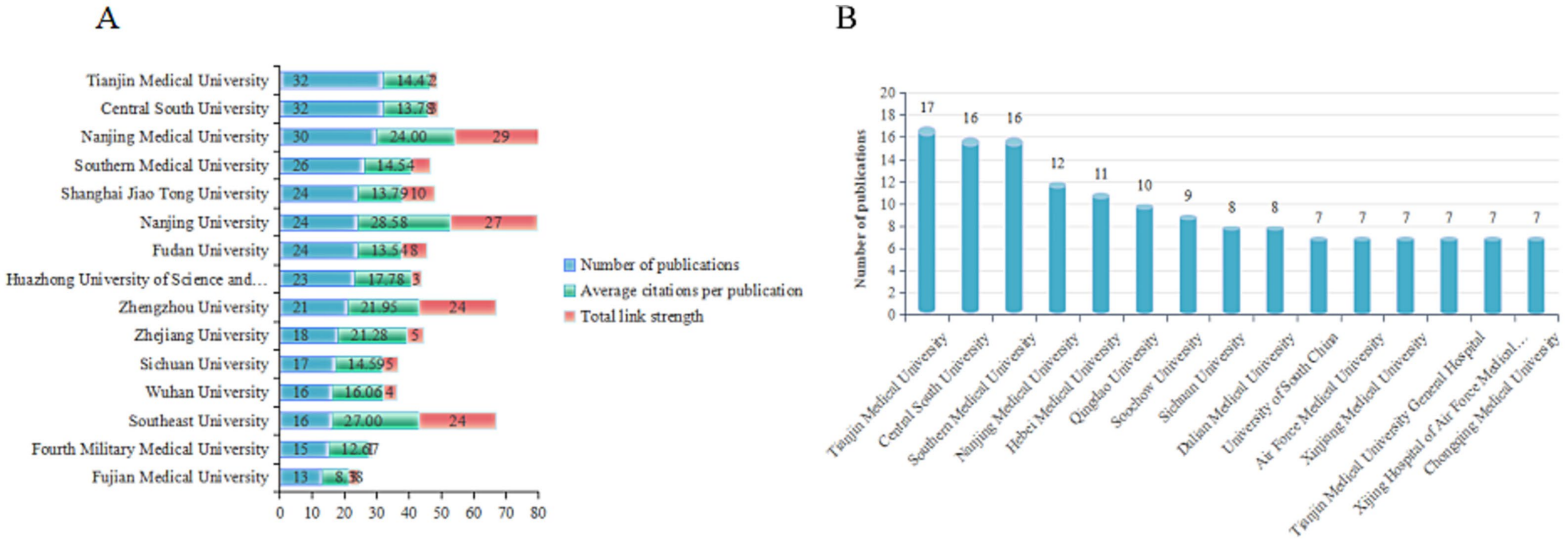
Distribution of SAE research institutions. **(A)** Top 15 institutional contributors in the WoS Core Collection database, presented by number of publications, average citations per publication, and total link strength. **(B)** Top 15 institutional contributors in the CNKI database, presented by number of publications.

A total of 822 institutions have issued research publications connected with SAE. According to analysis of these institutions (Figure 4A), based on the WoS Core Collection database, all top 15 institutions by publication quantity are situated in China; this thus underscores the leading function of Chinese medical universities inside this research domain. Among them, Tianjin Medical University and Central South University occupy the same top rank, each having 27 publications, even though their per-publication average citations and total link strength are at moderate levels. After them are Nanjing Medical University and Southern Medical University. Concerning academic influence, Nanjing University owns the highest per-publication average citations, which is 28.58. It is worth noting that Southeast University, even with fewer publications (16 publications), has a high per-publication average citation rate of 27.00; therefore this reflects the institution’s high-quality research and notable academic impact within this field. Figure 4B displays the publication situation of institutions in the CNKI database, where the top three are Tianjin Medical University (17 publications, Central South University publications), and Southern Medical University (16 publications). On the whole, Chinese medical universities show strong research abilities in the SAE field. Institutions such as Tianjin Medical University, Central South University, Nanjing University, and Southeast University take the lead in both research output and academic influence, and are gradually building research networks that center on these universities, hence this promotes continuous development of the field.

### 3.7 Keyword co-occurrence analysis

#### 3.7.1 The WoS database keyword analysis

**Figure 5:**
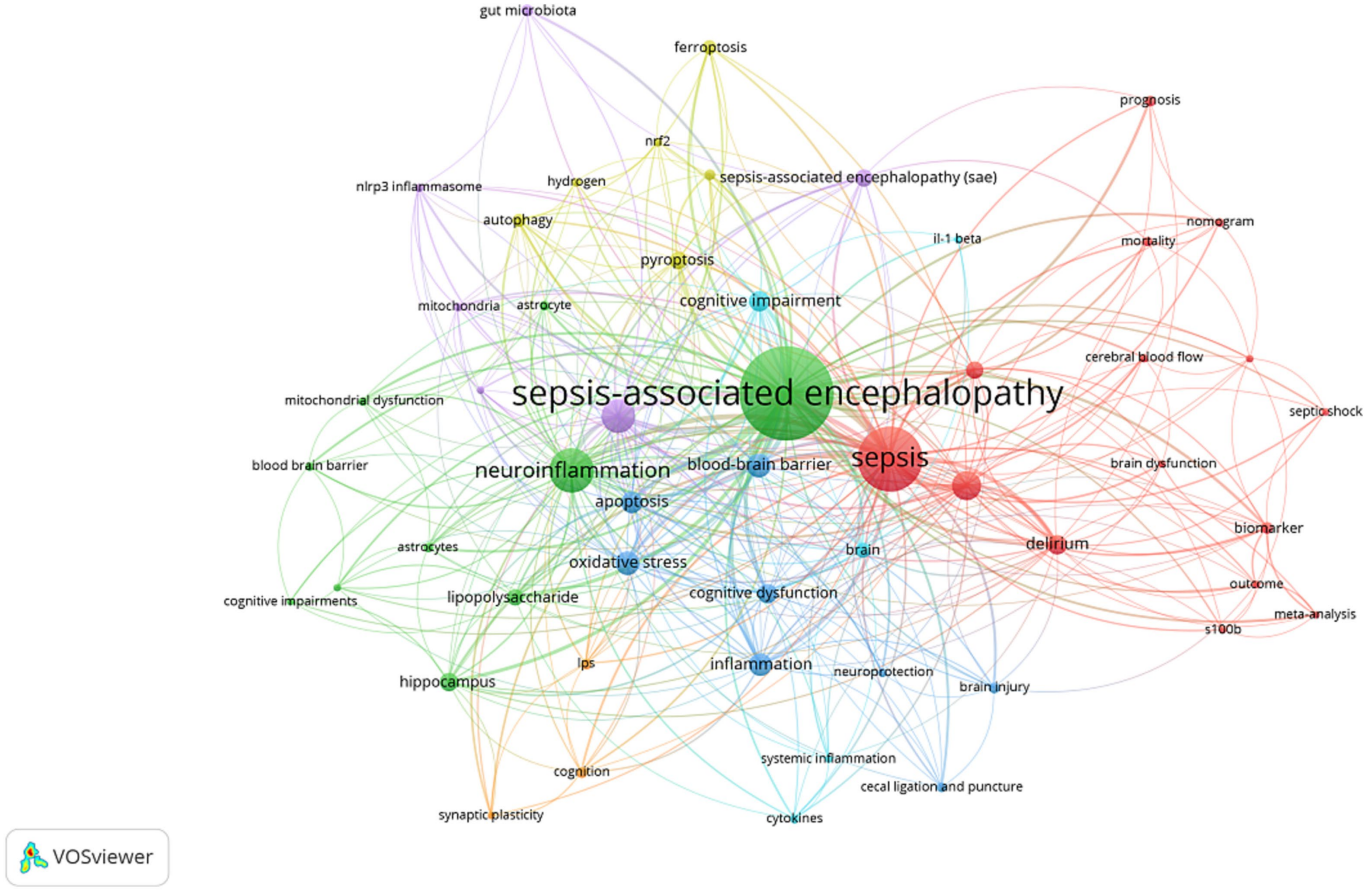
Network analysis of keyword co-occurrence in the WoS database.

In the WoS Core Collection database, the top 10 keywords ranked by frequency are: sepsis-associated encephalopathy (451 occurrences), sepsis (216 occurrences), neuroinflammation (155 occurrences), microglia (72 occurrences), cognitive impairment (62 occurrences), encephalopathy (58 occurrences), oxidative stress (41 occurrences), blood–brain barrier (41 occurrences), apoptosis (35 occurrences), and delirium (31 occurrences). The co-occurrence relationships among these keywords demonstrate the multidimensional characteristics of SAE research, as shown in Figure 5.

**Figure 6:**
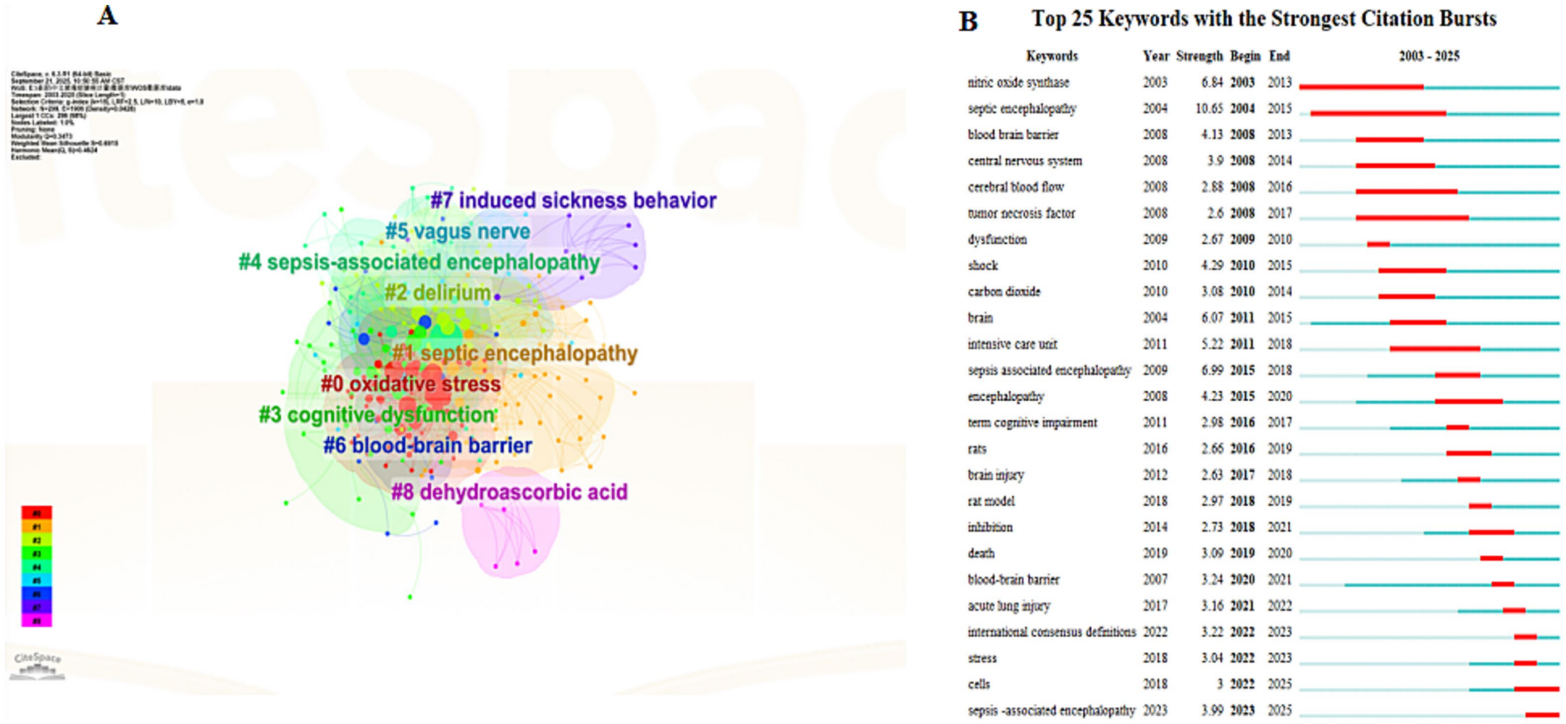
Clustering and burst detection analysis of keywords in the WoS database. **(A)** Clustering analysis of keywords related to SAE, with different colored clusters representing distinct research themes. **(B)** Top 25 keywords with the strongest citation bursts, showing the burst duration and strength of each keyword.

**Figure 7:**
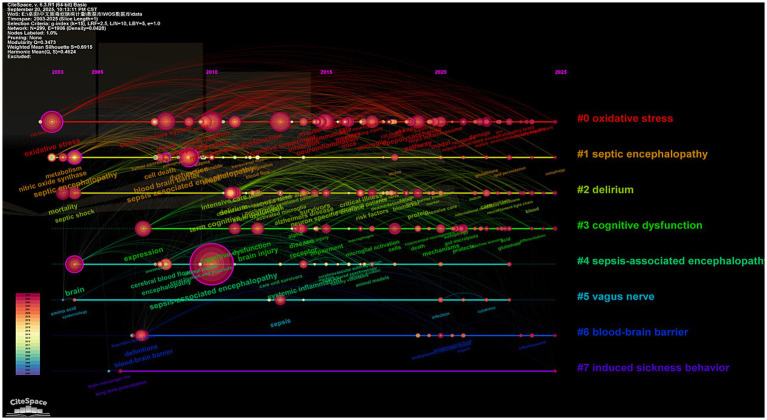
Timeline visualization analysis of keyword in the WoS database.

Cluster analysis of high-frequency keywords can achieve dimensionality reduction and integration of research directions, facilitating the rapid identification of research hotspots (Hou et al., 2022). Using CiteSpace software, researchers clustered and named keywords via the LLR method, ultimately categorizing the research field into nine main clusters (Figure 6A). The keyword clusters reveal three core research clusters: the pathological mechanism cluster (clusters #0, 3, 5, 6), which focuses on cellular-level pathological changes; the nervous system impact cluster (clusters #1, 2, 7), which centers on the blood–brain barrier and delirium, investigating the effects of sepsis on the central nervous system; the microenvironment regulation cluster (clusters #4, #8), which goes beyond the traditional “endogenous injury” perspective by introducing variable factors such as exogenous antioxidants, nutritional status, or environmental enrichment, exploring their regulatory effects on the disease process and providing a basis for subsequent “intervenable environment” strategies. The keyword cluster timeline visualization (Figure 7) depicts the development trajectory of various research themes. Node position reflects the origin time of a theme, node size represents research importance, and the number of nodes indicates research intensity, while connections between nodes show the evolution path of themes (Li et al., 2024). This timeline clearly shows that SAE research has evolved from the early “metabolism-barrier” binary exploration to an integrated research phase centered on the “mitochondria-ferroptosis-neuroinflammation” ternary interaction, framed by international consensus and targeting precise interventions.

Burst keyword analysis reveals high-intensity keywords, including: “nitric oxide synthase” (strength 6.84, 2003–2013), “septic encephalopathy” (strength 10.65, 2004–2015), and “tumor necrosis factor” (strength 2.6, 2008–2017), each with a burst duration of over 10 years. From a temporal perspective, research focuses a decade ago centered on terms such as “nitric oxide synthase,” “central nervous system,” “cerebral blood flow,” “tumor necrosis factor,” “shock,” and “carbon dioxide,” mainly concerning inflammatory mediators, brain metabolism, and systemic hemodynamics. In recent years, research frontiers have shifted to “international consensus definitions” (2022–2023), “sepsis-associated encephalopathy” (strength 3.99, 2023–2025), “cells,” “stress,” and “inhibition,” indicating that the research focus has transitioned from single inflammatory factors toward consensus standardization and integrated intervention directions focusing on multi-organelle stress and targeted inhibition, with continuous in-depth practical exploration (Figure 6B).

#### 3.7.2 The CNKI database keyword analysis

**Figure 8:**
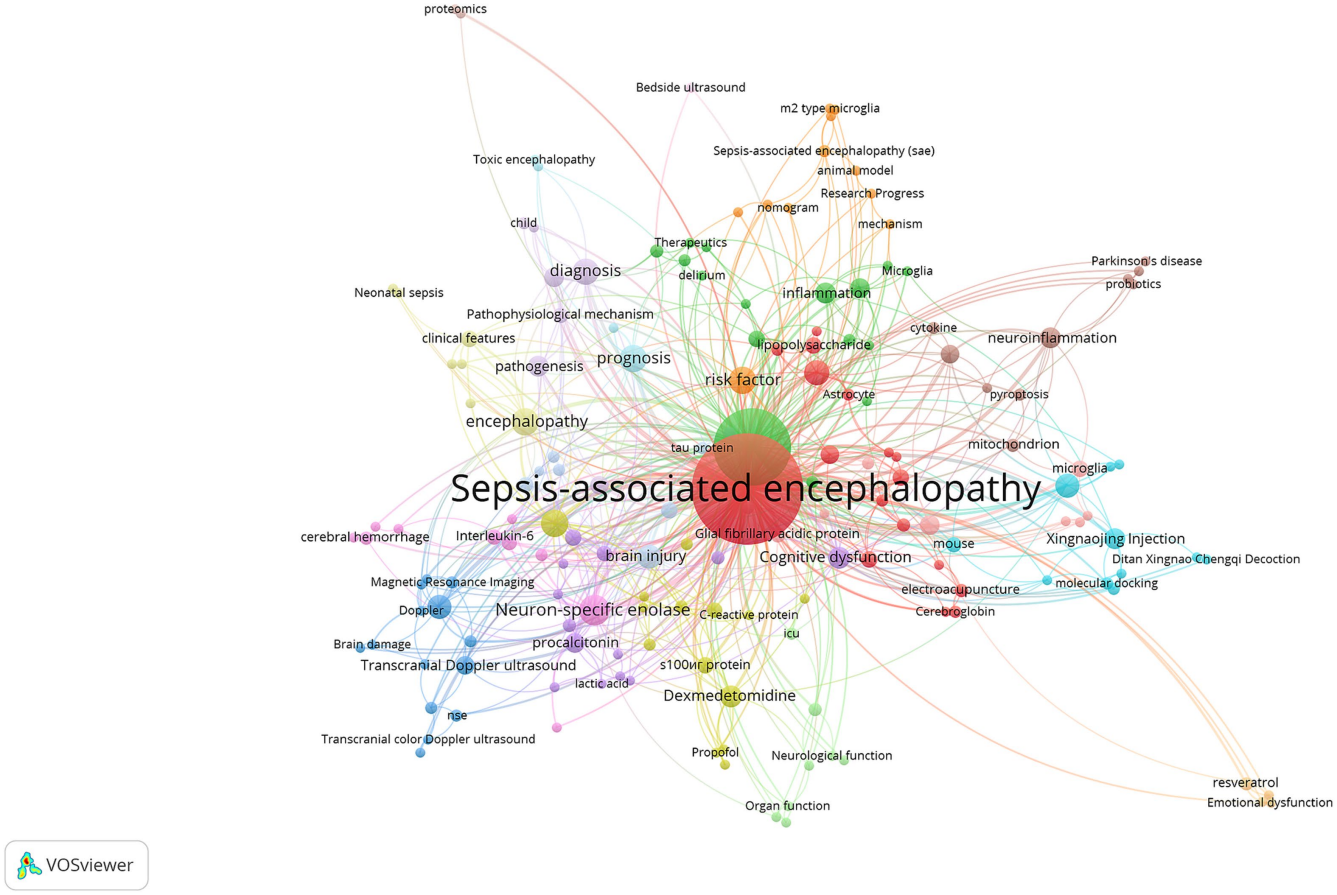
Network analysis of keyword co-occurrence in the CNKI database.

**Figure 9:**
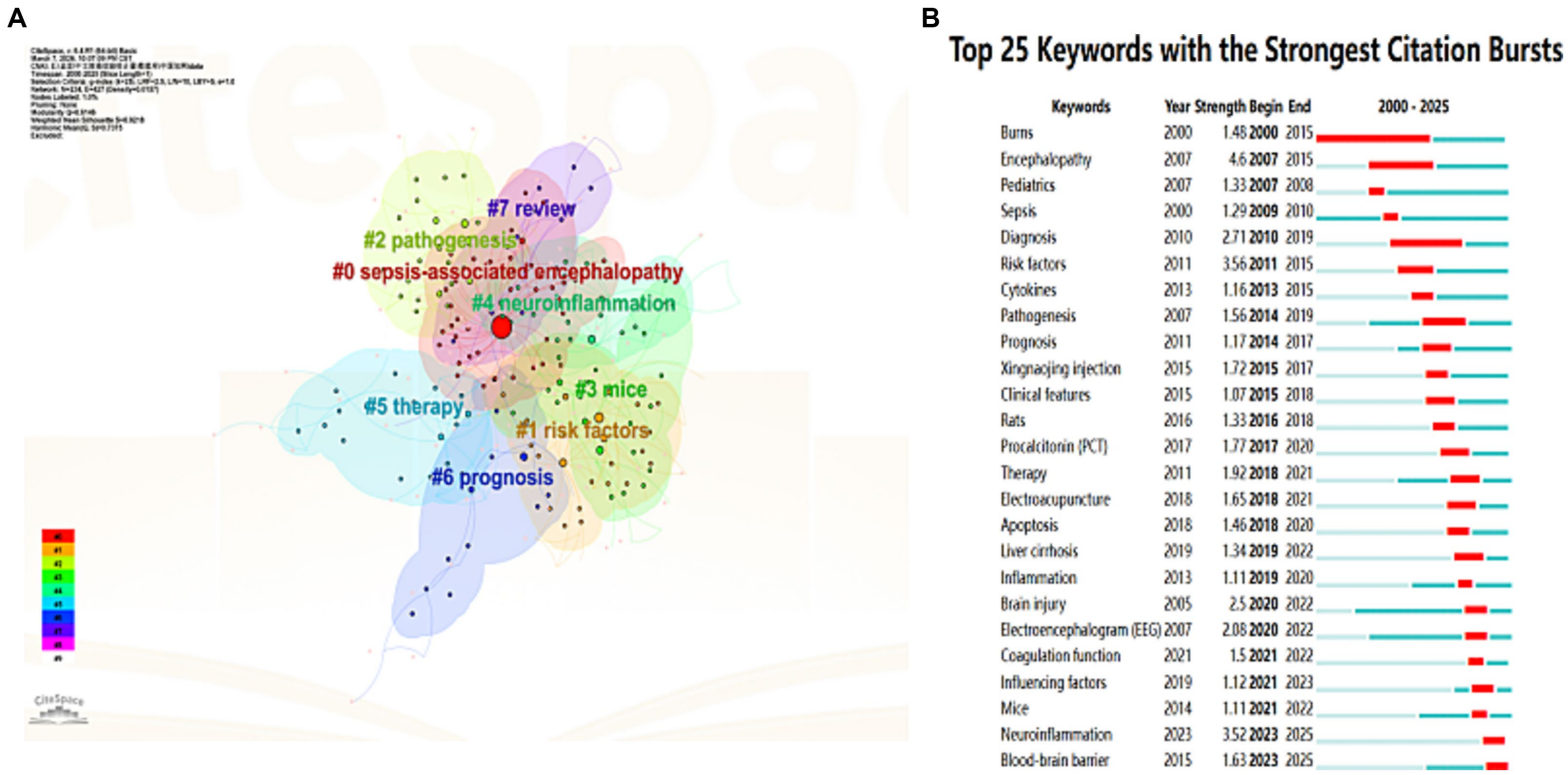
Clustering and burst detection analysis of keywords in the CNKI database. **(A)** Clustering analysis of keywords related to SAE, with different colored clusters representing distinct research themes. **(B)** Top 25 keywords with the strongest citation bursts, showing the burst duration and strength of each keyword.

**Figure 10:**
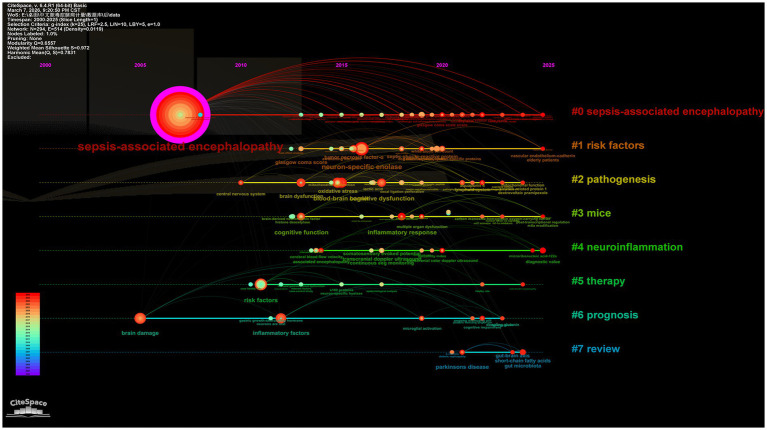
Timeline visualization analysis of keywords in the CNKI database.

In the CNKI database, the top ten most frequent keywords were as follows: sepsis-associated encephalopathy (250 occurrences), sepsis (122 occurrences), neuron-specific enolase (20 occurrences), children (16 occurrences), prognosis (16 occurrences), encephalopathy (15 occurrences), risk factors (15 occurrences), diagnosis (14 occurrences), blood–brain barrier (13 occurrences), and electroencephalogram (12 occurrences). The co-occurrence network of these keywords is shown in Figure 8. Our keyword cluster analysis further defined three key research themes for SAE, as presented in Figure 9A. Among them, the pathological mechanism cluster (#0, 1, 3, 4) clarifies the onset and development of SAE from three aspects: systemic inflammation, central immune response, and cytokine storm. The clinical assessment and prognosis cluster (#2, 5) focuses on the screening of high-risk children and quantitative prediction of outcomes, following the research route of high-risk population identification and prognosis evaluation. The knowledge integration and intervention cluster (#6, 7) is centered on treatment optimization and evidence summary. It shows that studies in China often combine systematic review methods with integrated traditional Chinese and Western medicine interventions, such as Xingnaojing combined with dexmedetomidine, to form evidence-based treatment plans and promote the integration of basic evidence and clinical application. We also plotted a keyword cluster timeline to show the dynamic evolution of research themes, as displayed in Figure 10. According to the timeline, studies retrieved from CNKI have gradually developed from early clinical observational descriptions to a comprehensive research system. At present, the core lies in the interaction between neuroinflammation and blood–brain barrier, accompanied by multi-target intervention strategies with traditional Chinese medicine, covering both pediatric and adult patients. Results from keyword burst analysis displayed distinct stage characteristics. During 2000–2010, research bursts mainly focused on burns, sepsis, and diagnosis. From 2011 to 2017, the focus turned to risk factors, cytokines, pathogenesis, and Xingnaojing. Between 2018 and 2020, bursts were observed in treatment, electroacupuncture, apoptosis, and procalcitonin. In the latest period from 2021 to 2025, neuroinflammation and blood–brain barrier became the most prominent burst terms.

In general, research hotspots have changed step by step. Early studies were limited to clinical observation, followed by mechanism exploration and Traditional Chinese Medicine (TCM) interventions represented by Xingnaojing. Later, the field developed toward targeted therapy such as electroacupuncture and biomarker detection. Now, the frontier has shifted to precise intervention strategies targeting the synergistic repair of neuroinflammation and blood–brain barrier function, as shown in Figure 9B.

## 4 Discussion

### 4.1 Global research trends

In this study, we systematically analyzed SAE-related publications from WOS and CNKI between 2000 and 2025, turning to VOSviewer and CiteSpace to tease out key patterns. Our analysis covered research collaboration networks, where things stand now, and where the field is headed. The data show global SAE publication output rising steadily over the past 25 years, with interest showing no signs of flagging. Although SAE research emerged relatively late compared with other sepsis-associated organ complications, such as sepsis-associated myocardial dysfunction and sepsis-associated acute kidney injury, it has developed rapidly and gained increasing academic attention in recent years, highlighting its unique and important value in the study of sepsis-associated complications (Zhang et al., 2022; Yao et al., 2023; Chen et al., 2024; Wang et al., 2025). Particularly after the COVID-19 pandemic emerged (de la Rica et al., 2020; Zanza et al., 2022), research in this area entered a phase of rapid expansion, with a sharp rise in relevant publications. This trend highlights the mounting attention SAE has received on a global scale. In regard to subject distribution, the field displays strong features of cross-disciplinary integration, with distinct research focuses across different disciplines. Neuroscience, for instance, centers on establishing causal links among inflammation, synaptic plasticity and consciousness disturbance. It also explores clinical translation strategies for vagus nerve stimulation and targeted optogenetic techniques. Immunology devotes efforts to clarifying mechanisms underlying microglial phenotypic switching, with the goal of developing targeted immunomodulatory agents. Pharmacology and pharmaceutical science, meanwhile, emphasize the design of nanomedicines capable of crossing the blood–brain barrier and building multi-target delivery systems based on the microbiota gut brain axis. Even though these disciplines share common platforms such as animal behavioral testing and molecular imaging, they each build multi-level interpretive frameworks from peripheral inflammation to central nervous system dysfunction. They do so along three major dimensions including neural circuits, immune signaling and drug delivery, together forming an integrated research system spanning molecular to behavioral levels. In the CNKI database, studies span multiple disciplines including emergency medicine, clinical medicine, neurology and pediatrics. This pattern suggests that domestic research places greater emphasis on the full clinical workflow including early identification, comprehensive treatment and prognostic assessment. It also reflects a clinical model characterized by multidisciplinary cooperation and integrated traditional Chinese and Western medicine.

### 4.2 Regional research characteristics and international cooperation

In terms of national publication output, China, the United States, and Brazil lead in SAE research. China accounts for the largest share. This pattern appears to reflect the heavy clinical demand during the COVID-19 pandemic, targeted policy support, sustained funding streams, and deep-rooted disciplinary infrastructure in China. In terms of international cooperation, China has established close cooperative relations with countries such as the United States, United Kingdom, and Germany, but the United States demonstrates a broader international cooperation network, possibly due to its globally leading academic influence, substantial scientific research resources, and open scientific communication environment (Luo et al., 2011; Wagner et al., 2015; Chen et al., 2023). Analysis of authors and institutions reveals Chinese researchers have been highly active in SAE studies. Xie Keliang has emerged as a key figure, with a large publication volume and relatively high total citations. Although China publishes more articles than any other country, the average citation per publication still falls behind that of several developed countries. This suggests the overall quality and global influence of Chinese research still have room to grow. Turning to institutions, all top 15 contributing organizations in WoS come from China. This reflects the strong standing of Chinese medical universities across the field. Tianjin Medical University and Central South University lead in total publications. Nanjing University and Southeast University perform well in terms of academic influence. Chinese institutions hold an obvious advantage in research scale, but further progress is still needed for depth and overall quality. CNKI findings follow a similar pattern. Tianjin Medical University, Central South University, and Southern Medical University also rank high in publication numbers. This supports the idea that major domestic medical universities play a central part in advancing SAE research.

### 4.3 Evolution of research hotspots

The most frequently used keywords in both WoS and CNKI include “sepsis-associated encephalopathy,” “sepsis,” “encephalopathy,” and “blood–brain barrier.” Keyword clusters from WoS indicate three major research directions. The first explores pathological mechanisms including mitochondrial dysfunction (Fu et al., 2024; Zhang et al., 2024), ferroptosis, and microglial activation (Moraes et al., 2021; Hu et al., 2023; Huo et al., 2023). The second focuses on the influence of sepsis on the nervous system, with emphasis on blood–brain barrier injury and mechanisms linked to delirium (Gao and Hernandes, 2021; Peng et al., 2021). The third covers microenvironment regulation, including exogenous antioxidants, nutritional status, and international consensus definitions. The field has expanded from the early framework of metabolism and barrier function to include multi-organelle interactions and unified international standards (Kanczkowski et al., 2016; Zhao et al., 2020).

CNKI clusters show four major research themes. These themes include the interaction between neuroinflammation and blood–brain barrier, early warning and prognosis for high-risk pediatric groups, multi-target interventions of TCM such as Xingnaojing and electroacupuncture, and repair strategies based on multi-omics. It should be explicitly clarified that this study is a bibliometric analysis, which only reflects the research hotspots and output trends in the field, and does not verify the clinical therapeutic efficacy of interventions such as electroacupuncture and TCM.

Burst analysis from WoS displays a clear shift in research focus. Around ten years ago, most studies focused on basic mechanisms such as “nitric oxide synthase”, “cerebral blood flow”, “tumor necrosis factor”, and “carbon dioxide.” In recent years, topics have become more clinical and intervention oriented, including “international consensus definitions”, “sepsis-associated encephalopathy”, “cells”, and “inhibition.” Research has moved from analysis of single inflammatory factors to combined interventions that include standardized guidelines, multi-organelle responses, and targeted inhibition. CNKI burst keywords also show a three-stage developmental trend. Early studies focused on “risk factors”, “cytokines” and “pathogenesis.” Later research turned to “treatment”, “electroacupuncture”, and “apoptosis.” The latest studies focus on “neuroinflammation” and “blood–brain barrier.” Domestic research has gradually changed from basic mechanism exploration to clinical translation. Many current studies combine TCM interventions with precise barrier repair, forming an integrated system that connects basic and clinical research.

Future research should focus on the core relationship between neuroinflammation and blood–brain barrier. Methods such as single-cell multi-omics (Lu et al., 2025; Garduno et al., 2023), organoid chips, and vagus nerve regulation can be used for further exploration. More evidence-based evaluations and mechanism studies are also needed for interventions such as Xingnaojing and electroacupuncture. These efforts can help develop more accurate combined treatment plans that integrate traditional Chinese and Western medicine. Further studies can also promote the update of international consensus. Research can cover the whole process from biomarker based early warning to intervention and prognosis. This will help the field move from mechanistic research toward real clinical application and transformation.

### 4.4 Advantages and limitations

This study integrates English and Chinese literature from the WoS Core Collection and CNKI. With a 25-year timespan, it captures the full progression and recent advances of SAE research worldwide. The dual-database design avoids bias seen in studies limited to one database or one language. We examined publication trends, disciplines, countries, institutions, collaborations, and keyword dynamics and also grouped the development of SAE research into three stages. These stages show how the field has shifted from basic mechanistic studies to targeted clinical strategies. We further compared global and Chinese research. Global work centers on basic mechanisms. Research in China focuses more on clinical practice and combined traditional Chinese and Western medicine. All results come from carefully processed and checked data. They offer dependable guidance for future work in this field.

Nevertheless, this study had several limitations. First, our data were only retrieved from the WoS Core Collection and CNKI; relevant studies published in other databases were not included, which may have introduced some bias into the analytical results. Second, when we used average citations per paper to evaluate research quality, we recognized several potential confounding factors: older publications tended to have more citations, WoS and CNKI differed in database coverage and language of publication, and citation counts could also be affected by self-citations and the size of the research field. These factors may have reduced the comparability of citation-based metrics. Third, recently published high-quality publications could not be fully identified in burst detection analysis because of their short citation period. Finally, keyword clustering was performed mainly based on word frequency; the deeper semantic connections between some complex concepts still needed further exploration and verification with more advanced text mining approaches.

## 5 Conclusion

In summary, this study uses 25 years of dual-database data to trace how SAE research has evolved, what now dominates the field, and where it is heading. It maps the global picture, disciplinary spread, and key shifts, offering guidance for future research and clinical work. Going forward, the field should focus on the neuroinflammation-blood–brain barrier link, strengthen cross-discipline collaboration, and drive original innovations and targeted interventions that improve patient outcomes.

## Data Availability

The original contributions presented in the study are included in the article/supplementary material, further inquiries can be directed to the corresponding author.
